# Supplementary material for: Are carbon emissions trading and green financial instruments synergistic? -Comprehensive quantitative research based on content analysis
Source: PLoS One. 2024 Mar 7;19(3):e0298601. doi: 10.1371/journal.pone.0298601 (PMC10919601; doi:10.1371/journal.pone.0298601)
Supplement: S1 Data — (ZIP) [file pone.0298601.s001.zip › Data/地级市Z_F工作报告.zip!/╔╜╬≈/╦╖╓▌╩╨/└┤╫╘╣1⁄2╓┌║┼í╛┬φ┐╦╩2╛▌═°í┐_2017.docx]

各位代表：

　　现在，我代表市人民政府向大会报告工作，请予审议，并请市政协委员和其他列席人员提出宝贵意见。

　　一、2016年工作回顾

　　2016年，是我市经济异常艰难、下行触底的一年，也是全市上下砥砺前行、孕育希望的一年。面对严峻复杂的宏观经济形势和主要经济指标持续下滑的困难局面，我们认真贯彻落实党中央、国务院和省委、省政府一系列决策部署，紧扣市委提出的“两大任务”工作主题，坚持以经济建设为中心，凝心聚力，强化调度，精准施策，推动经济止跌趋稳、稳中向好，实现了“下半年好于上半年，为2017年进一步好转奠定坚实基础”的目标。

　　全年地区生产总值完成918亿元，同比增长4.2%，比上半年提高3.8个百分点；规上工业增加值完成213.1亿元，同比增长2.7%，比上半年提高5个百分点；一般公共预算收入完成49.1亿元，同比下降9.5%，比上半年收窄19.5个百分点；固定资产投资完成637.7亿元，同比下降31.9%；社会消费品零售总额完成291.1亿元，同比增长7.7%；城镇居民人均可支配收入28989元，同比增长5.4%；农村居民人均可支配收入11478元，同比增长6.1%。约束性指标中，万元地区生产总值能耗下降幅度、二氧化碳排放量下降幅度、用水量下降幅度，二氧化硫、化学需氧量、氨氮、氮氧化物、烟尘、粉尘减排幅度，劣V类水体比例，农村贫困人口脱贫人数、城镇棚户区住房改造数量等指标均完成年度任务。总体来看，我市经济正在发生一些标志性、转折性、趋势性变化，经济发展的方向更加明确，稳步向好的基础不断巩固，干事创业的氛围日益浓厚。

　　回顾过去一年，能够取得这样的成绩，实属不易。这些成绩，是在世界金融危机余波未息、全球经济持续低迷、国际环境非常不利的形势下取得的。这些成绩，是在我国经济发展进入新常态，速度变化、结构优化、动力转换，全省能源主导型经济低位徘徊的形势下取得的。这些成绩，是在我市经济连续30个月持续下滑、上半年9项主要经济指标排全省末位的被动局面下取得的。这些成绩的取得，主要得益于我们认真学习贯彻习近平总书记系列重要讲话精神和治国理政新理念新思想新战略，落实省委提出的“一个指引、两手硬”重大思路和要求；得益于市委、市政府紧紧抓住全省创优发展环境和煤炭市场好转的有利机遇，以经济建设为中心，采取了一系列果断而有力的措施；得益于全市广大干部群众团结努力、积极进取，形成了推动发展的强劲合力。这些成绩的取得，为全市经济在新的一年里进一步摆脱困境、持续好转创造了条件，奠定了基础；为全市人民在新的一年里进一步加快发展、拼搏进取提振了信心，增添了动力！

　　一年来，我们主要抓了以下八方面工作：

　　（一）努力促进经济企稳回升。我们深入分析了“十二五”以来我市经济“大起大落”的深层次原因，果断采取了遏制经济继续下滑、保持低位企稳的稳控措施，主要办法是突出抓了“六个发力点”：提高重点工程开工率，帮助优势企业增产增效，科学释放煤炭先进产能，促进新建企业投产达效，推动三产服务业提质增效，保持农业农村经济稳定增长。特别是紧紧抓住重点工程开工建设和优势企业增产增效这两个关键点，在全市上下扎实有效开展了“双200集中帮促”行动。坚决贯彻省委、省政府万名干部入企服务的要求，市县两级共有759名干部深入企业蹲点包扶，先后解决了200多个突出问题，有效地调动了企业的积极性。到9月底，工业品出厂价格指数结束了连续39个月负增长，工业经济扭转了连续20个月负增长的态势。行动中对新建未开工的204项重点工程进行了集中攻坚，推动152个项目当年开工。市政府建立了每月“四个调度会”的制度，加强对经济形势的深入研判，解决发展中的突出问题。

　　（二）狠抓经济转型和结构调整。在经济结构调整中，大力发展非煤产业，从煤以外积极寻找战略突破点。新能源电力产业又有6家风电和光伏电厂建成投产，装机规模为46.8万千瓦，全市新能源电力装机规模达到406万千瓦，排在全省前列。晋北（朔州）现代煤化工基地建设有了新进展，签署了阳煤、国新能源总投资37亿元的低阶煤清洁高效利用多联产项目合作协议。三元炭素碳纤维、中美新能源现代煤化工中试、韩国爱德林垃圾处理等一批高科技项目启动。积极推动新材料、陶瓷、装备制造、食品、医药、电动汽车等产业发展。非煤产业工业增加值占规模以上工业比重达到32.5%，比上年提高6.3个百分点。服务业成为拉动经济增长的新动力，增加值占经济总量的比重达到50.8%。旅游业发展也呈现好的势头，旅游业收入完成160.7亿元，比上年增长20.3%。

　　（三）积极推进城镇化建设。全市组织实施了城市人居环境改善“四大工程”，完成投资119亿元。保障性住房建成10098套，完成投资30.32亿元。农村危房改造和抗震住房加固7670户，完成投资1.3亿元。户籍制度改革取得了新进展，又有1万多农村户籍人口转为城市人口。全市城镇化率达到54.2%，比上年提高1个百分点。市城区继续推进七里河综合治理、“三路四桥”、恢河大桥等35项重点工程，完成投资26亿元。进一步扩大城市绿化，新增绿地面积55万平方米，绿化覆盖率达到42.6%。进入冬季，围绕解决“脏乱差”问题，组织开展了为期100天的市城区市容市貌整治“五乱”和卫生清洁两个“专项行动”。行动中集中清理整治各类“五乱”问题3000多个，市容市貌发生了明显变化。“五城联创”取得了新的成绩，成功创建了全国双拥模范城。

　　（四）促进农民收入稳定增长。粮食总产达到25.4亿斤，比上年增长15.5%，成为历史上第二个高产年。国家草牧业试验试点和“粮改饲”工作有了新进展，完成耕地种草60万亩。全市奶牛存栏18.7万头，肉羊饲养量591.5万只。畜牧业产值达到55.9亿元，占农业总产值的46.1%。奶牛存栏量、鲜奶产量、肉羊年出栏量、人均畜产品年占有量、农民年人均畜牧业纯收入以及畜牧业占农业总产值比重六项草牧业发展指标继续保持全省第一。积极培育新型农业经营主体，各级各类家庭农场和农民专业合作社达到了8500多个。按照“六个精准”和“五个一批”的要求，认真组织实施十大脱贫攻坚工程，完成了59个村、22961名贫困人口的减贫任务。

　　（五）进一步推动改革开放。大力推进供给侧结构性改革，落实“三去一降一补”任务，关闭了7座煤矿，退出了1100万吨产能，为全省作出了贡献；房地产去库存减少存量71.5万平方米。围绕“整合、扩区、改制、调规”四大任务，开发区改革创新发展工作全面展开，“三化三制”内部改革也做了大量准备工作。农村土地承包经营权确权登记颁证工作接近尾声，农村集体产权制度改革试点工作全面启动。金融创新有了新突破，市政府搭建了四大融资平台，建立了总规模为200亿元的城市发展基金和经济发展基金，大大增强了政府公共投资能力、财政保障能力和企业融资能力。商事制度改革见到了新的成效，新增各类市场主体近1.2万户。先后组织开展了北京、山东、浙江、重庆、厦门、深圳等点对点的招商引资活动，签约引资项目172个，投资总额达1651亿元。

　　（六）加快生态文明建设步伐。持续推进造林绿化，完成新造林17.4万亩。深入开展大气污染防治，推进实施了电力行业超低排放改造、淘汰燃煤小锅炉和黄标车、清洁燃煤替代等重点任务。水污染防治、生态修复治理和地质灾害治理取得新的成效。

　　（七）高度重视民生和社会事业。在地方可用财力大幅减少的情况下，优化财政支出结构，确保民生投入稳步增长。就业创业工作完成了当年目标任务，新增城镇就业21500人。社会保障体系建设进一步完善，机关事业单位养老完成了并轨，大病保险制度实现了全覆盖，全民参保登记率达到了96.3%，新农合医保参保率达到了99.4%。教育事业有了新发展，朔城区、山阴县、应县顺利通过国家义务教育均衡发展评估验收，全市高考二本以上达线人数13828人，达线率41.7%，高出了全省平均水平6.2个百分点。全市乡镇卫生院、社区卫生服务机构、村卫生室实现了基本药物制度全覆盖。国家公共文化服务体系示范区建设获得了文化部和财政部授牌。竞技体育事业取得了新的成绩。启动了大西高铁、右平高速等一大批事关民生的重大项目，开通了“朔州号”旅客列车，右玉通用飞机场建设项目获得国家有关部门批准。市城区集中供热体制改革，理顺了政府与企业、运营企业与热源企业、热源企业与热源企业之间的利益关系，扩大了供热面积，提高了服务质量。全面落实安全生产责任，强化重点行业领域专项整治，各类安全生产事故起数、死亡人数与“十二五”期间平均水平相比，分别下降27%和25%。加强国防后备力量建设，积极开展双拥工作。发展妇女儿童、老龄和残疾人事业，信访、民族、宗教、外事、侨务、地震、气象、档案、史志等各项工作都取得新成绩。

　　（八）加强政府自身建设。市政府制定出台了《关于加强政府自身建设的意见》，提出要不断加强思想政治建设、强化全心全意为人民服务的宗旨意识、提高履行职责的能力和水平、大力弘扬为民务实的优良作风、加强法治政府建设、深入推进党风廉政建设和反腐败斗争六项具体要求。深入推进“放管服效”改革，进一步落实了“两集中、两到位”的要求，市级公共资源交易全部进入了交易平台。认真落实全面从严治党主体责任，扎实开展“两学一做”学习教育，持续推进政府系统党风廉政建设和反腐败工作。自觉接受人大和政协监督，支持各民主党派、工商联、无党派人士参政议政，办理人大代表建议106件、政协委员提案95件。

　　各位代表，过去一年取得的成绩，是省委、省政府和市委正确领导的结果，是市人大、市政协监督支持的结果，是全市人民齐心协力、共同奋斗的结果。在这里，我代表市人民政府，向全市人民和所有关心支持朔州发展的社会各界人士，表示衷心的感谢和崇高的敬意!

　　当前，我市经济虽然出现了积极向好的态势，但发展的基础还不牢，一些制约经济发展的深层次问题并没有根本解决，整体上仍处于建市以来最困难的时期。发展中存在的短板和问题主要是：一是经济总量不大。地区生产总值在千亿元左右徘徊，在全省11个地市中排第9位，在全国297个地级以上城市中排第220位。二是产业结构不优。“一煤独大、一企独大、一股独大”，非煤产业、高新技术产业和民营经济发展都很不充分。三是发展后劲不足。大工程大项目、好工程好项目少，新兴产业发展不足，创新驱动力度不大。四是城镇化发展水平不够高。基础设施建设欠账较多，公共服务配套不足。五是经济管理比较粗放。区域管理水平不够高，现代企业制度还不完善。特别是煤炭产业的规模与对地方的贡献不相称。六是发展环境有待于进一步优化。仍然存在办事拖拉、互相扯皮、效率不高的问题。一些地方和部门服务企业、服务项目和服务群众的工作做得很不够。少数地方重大项目建设中阻工问题时有发生。七是对外开放水平不够高。近年来引进的新兴产业、先进制造、加工贸易企业不多也不大，资源输出与项目

　　引进很不匹配。这些问题，既是我市经济发展的短板，也是制约发展的“瓶颈”。我们一定要高度重视、勇于面对，以对历史和人民负责的态度，以敢于啃硬骨头的斗争精神，认真加以解决，努力走出朔州转型发展的新路子！

二、2017年工作总体要求

　　2017年是实施“十三五”规划的重要一年，是全面贯彻省第十一次党代会、市第六次党代会精神的起步之年，也是我市经济克服困难、走出困境的关键之年。今年政府工作的总体要求是：深入学习贯彻习近平总书记系列重要讲话精神和治国理政新理念新思想新战略，统筹推进“五位一体”总体布局，协调推进“四个全面”战略布局，全面贯彻落实党的十八大和十八届三中、四中、五中、六中全会，以及省第十一次党代会和市第六次党代会精神，按照省委“一个指引、两手硬”重大思路和要求，坚持稳中求进工作总基调，践行“五大发展”新理念，主动适应经济发展新常态，深入贯彻落实市委“两大任务”和“五条新路”的总体部署，紧紧扭住“经济增长、产业转型、改革突破、改善民生”四个关键，全面提升经济发展质量、人民生活水平和整体发展形象，加快推进全市经济、政治、文化、社会和生态文明建设，以优异的成绩迎接党的十九大胜利召开。

　　主要预期指标是：地区生产总值增长5.5%，在实际工作中争取更好；规模以上工业增加值增长4%左右；一般公共预算收入增长8%；全社会固定资产投资增长目标，待省里根据新的统计口径研究设置后，再结合我市实际研究确定；社会消费品零售总额增长7%左右；城乡居民人均可支配收入分别增长6%左右和6%以上；居民消费价格涨幅控制在3%左右；城镇登记失业率控制在4.2%以内；约束性指标完成省定任务。

　　确定今年的发展目标，主要考虑了四条原则：一是稳中求进的原则。2014年地区生产总值的增幅是4.5%，2015年是-2.3%，2016年是4.2%。今年预测新的增长要素安排了5.5%的增幅，是2013年以来的最好水平，是一个用力跳才能实现的目标。二是积极进位的原则。在全省发展格局中由后进进入中等水平，有条件的县区和有条件的指标争取进入全省先进行列。三是实事求是的原则。预期目标定位在全省平均水平，显然不是一个先进目标，主要考虑了我市主要经济指标连续三年下滑的实际。四是有利转型的原则。既要考虑稳增长，也要考虑调结构，为供给侧结构性改革和产业转型升级留有一定余地和空间。

　　实现今年的发展目标，虽然难度很大，甚至可能遇到意想不到的困难和问题，但我们充满信心。信心来自于这样几个方面：第一，我们的信心来自于中央稳中求进的工作总基调。中央经济工作会议为实现中高速增长提出了一系列新的重大政策措施，特别是积极有效的财政政策和稳健中性的货币政策对我们极其有利。第二，我们的信心来自于省委、省政府治晋理政的重大思路。以省第十一次党代会为标志，山西的改革发展翻开了崭新的一页，“五个山西”的重大思路将进一步激发全省干事创业的热情，加快经济结构调整、促进经济转型将成为今年山西发展的主旋律。省内环境对我们极其有利。第三，我们的信心来自于全市已有的发展基础。朔州建市28年来形成了较好的物质基础，“十二五”期间，全市固定资产投资累计完成3600多亿元，这是一个较大的增长动力。第四，我们的信心来自于我市潜在的发展优势。应该看到，我市产业转型和结构调整的条件和时机已经成熟，农业、三产服务业、文化旅游业等新的动能正在形成，区域特色和综合优势正在显现。只要我们进一步解放思想，更新观念，把发展的思路更拓展一些，就一定会在共克时艰中突破困境，圆满实现经济社会发展目标，向全市人民交出一份满意的答卷！

　　围绕实现新的发展目标，在全市经济和社会发展中，我们要紧紧扭住“经济增长、产业转型、改革突破、改善民生”四个关键。第一个关键，经济增长。当前朔州经济发展中既有质量不高的问题，也有总量不大的问题。我们要在发展中把结构性和总量性的问题统筹起来考虑，既要强调质量，又要保持合理的增长水平。第二个关键，产业转型。推动经济转型对朔州来说是一项十分急迫的任务，确实等不得、慢不得。在煤炭产业可支撑的情况下，抓住机会转。如果失去了煤炭支撑，我们将面临更大的困难，承受更大的代价。第三个关键，改革突破。当前，经济社会发展中存在的一系列矛盾问题都必须用改革和创新的办法去解决。第四个关键，民生改善。民生改善是我们加快发展的最终目标，就是要让人民群众在发展中获得实实在在的成果。

　　我们力争经过几年的艰苦努力，到“十三五”末，使朔州经济社会发展由量变到质变，实现三个全面提升：第一，全面提升经济发展质量。我们要紧紧围绕转变经济发展方式、提升经济发展质量，一手抓传统产业升级改造，一手抓非煤产业特别是战略性新兴产业发展。非煤产业占工业增加值的比重每年要增加2个百分点，由“十二五”末的近27%提高到37%，战略性新兴产业占比明显提高，科技贡献率明显提升，资源能源消耗明显下降。第二，全面提升人民生活水平。到2020年不仅要使全市地区生产总值翻一番，也要使城乡居民人均收入翻一番，与全国人民一道迈入小康社会。发展中我们一定要围绕共享发展的目标，把握好三个速度：城乡居民收入增长要高于地区生产总值增幅、农村居民收入增幅要高于城镇居民收入增幅、贫困县的经济增长幅度要高于非贫困县的增长幅度。第三，全面提升整体发展形象。树立新的资源观，深刻认识形象也是无形资源。形象好了，美誉度高了，就会“栽下梧桐树，引得凤凰来”。这里所说的“形象”，不是表面的，而是由内而外生发出的美好形象。

　　整体形象如何提升？我们要立足于朔州现有的发展基础和比较优势，努力建设并展示六个新形象：一是绿水青山的生态形象。继续深入持久地开展大规模的植树造林，实施碧水蓝天建设工程，使朔州的山更绿、水更清、天更蓝。二是牛羊遍地的“草原形象”。把发展草牧业作为战略取向，在全市广大农村唱响“念牛经、发羊财”的“主旋律”，构建横向到边、纵向到底的草牧业全产业链。三是能源产业的现代形象。全面提高能源产业的管理水平、技术水平和装备水平，进一步推动煤炭产业安全发展、绿色发展、清洁发展。四是历史厚重的文化形象。在整个中华民族发展史上，雁门关地区始终处在民族融合的前沿和民族文化的交错地带，历史文化资源极其厚重。我们要深入挖掘、充分展示朔州的边塞特色，大力发展文化旅游产业，全面提升朔州的文化软实力。五是宽厚包容的开放形象。宽厚包容是朔州人比较典型的人文特征，我们要大力弘扬这种开放精神，让越来越多的人认识朔州、走进朔州、投资朔州。六是民生富裕的安康形象。朔州资源禀赋丰富、地理位置独特，历史上也是一个很“养人”的地方。在今后的发展中，全面建成小康社会的目标，就是让人民群众更加富裕、更加安康。

　　这里提出的美好形象建设，紧紧围绕经济转型这个中心，包含了生态文明建设、农业结构调整、能源绿色发展、提升文化软实力、加快对外开放步伐和提高人民生活水平这六个战略支点，是习近平总书记提出的“五大发展”理念在朔州的具体实践。我们决不能把形象建设搞成形象工程

　　，而是要把六个形象建设作为朔州未来发展的转型工程、富民工程和战略工程。我们构想，未来的朔州，绝不仅仅是一个煤都、电都、奶都、瓷都，而是一个产业多元、发展强劲、特色鲜明、充满生机和活力的现代魅力城市！

三、2017年的工作任务

　　2017年要重点做好十个方面工作：

　　（一）全力推动经济稳定增长。我市经济出现的许多积极变化来之不易。要巩固这种好势头，走出困难局面，还需要爬坡过坎，付出更大的努力。今年经济工作的基本任务是继续推动全市经济稳步向好，尽快进入合理增长区间。

　　发挥投资对经济增长的关键作用。今年全市安排了省市重点工程571项，总投资3600多亿元，其中当年完成投资610亿元。这些工程涵盖了农林牧水、交通、电力、煤及煤化工、轻工医药、材料工业、商贸物流、城建环保、旅游、保障性安居工程及房地产、科教文卫社会事业以及重大基础设施等13个重点领域。我们要举全市之力，突出抓好对经济发展具有全局意义的50个重大项目。特别是市城区基础设施全面配套工程，开发区扩区升级工程，飞机场、大原客专和右平高速等重大基础建设工程，两条电力外送通道工程，大型火电和新能源电力建设工程，七里河综合治理和桑干河生态修复综合治理工程，右玉全域旅游等文化旅游产业开发工程，文化教育卫生等公共文化设施工程，以及三元炭素碳纤维中试项目、右玉甘草酸项目等高新技术项目。这些项目呈现出明显的转型导向：产业投资在总投资中的比重达到了40%；战略性新兴产业投资在产业投资中的比重达到了18%；民间投资在各类投资中的比重达到35%。

　　支持和发展实体经济。实体经济是市场经济的主体，也是国民经济的基石。我们一定要在全社会牢固树立实体经济是发展之母、实体经济真正创造财富的战略思想，把发展经济的工作重心和注意力放到关注实体经济上，把支持实体经济的政策措施落实到实体企业中。落实减税降费政策，努力减轻企业税费负担。努力引导实体经济加强管理，节能降耗，降低成本，提高企业效益。要加强对实体经济的支持和服务，帮助企业解决自身难以解决的问题。该政府出面的政府要出面，对一些重大疑难问题，该领导出面的领导要出面，使企业在遇到困难时能够感受到来自各方面的帮助支持。要扎实开展干部入企帮扶活动，在市县经信部门设立服务企业办公室，有计划、有步骤地组织各级领导干部深入到重点企业帮助和指导，逐步形成长期的帮扶机制。要深入开展“朔州企业家投资在朔州”活动，鼓励本土创业者在本地放手投资，兴办实体经济，实现企业家的“二次创业”。

　　提高煤炭产业标准化、规范化、现代化建设水平。在现代化建设上，紧紧围绕安全质量标准化矿井建设，瞄准国际一流的现代化矿井建设，抓投入、抓技术、抓关键设备，增加有效投资，完善硬件建设，大幅度提高煤炭现代产能建设，使先进产能占比达到70%以上，高标准示范性矿井数量达到45个以上。在提升产业管理水平上，我们要把今年作为煤炭产业管理水平提升年，切实加强企业的技术管理、劳动管理、现场管理、信息化管理以及后勤管理。要鼓励和引导企业，在发展中既要讲效益，又要讲贡献，正确处理好国家、地方和企业之间的关系。国有大型煤炭企业既要抓好煤炭产业的发展，又要推动产业转型，积极发展非煤产业。在提升产业发展水平上，深入贯彻国家煤炭供给侧结构性改革和“三去一降一补”政策，坚定不移走集约、节约、延伸、增值、绿色的内涵式发展道路。要从根本上转变煤炭产业发展过程中存在的粗放式、扩张式，甚至是掠夺式的发展方式。

　　提升县域经济发展水平。县域经济是国民经济发展的基本单元，在经济社会发展中处于举足轻重的战略地位。县域经济稳步向好，是全市经济稳步向好的重要基础。各县区要立足资源优势和产业基础，打造各具特色的产业发展模式，不断壮大县域经济。朔城区、平鲁区要促进煤电产业提质增效，培育壮大战略性新兴产业，提升现代服务业发展水平。山阴县要促进现代煤化工项目落地，扩张乳品产业。怀仁县要重点抓好陶瓷产业上档升级，特别是要把肉羊做成大产业。应县要在发展现代农牧业的同时，扩张和提升陶瓷产业，特别是要把应县木塔的文章做足做好。右玉县要打好右玉精神和生态旅游两大品牌，全力推动国家级全域旅游示范区建设。

　　（二）大力促进经济转型升级。

　　培育壮大现有的非煤产业和新兴潜力产业。要抓好具有一定产业基础的高档陶瓷、先进装备制造、新材料、新能源、生物制药、特色农产品加工、文化旅游、现代物流业等新兴潜力产业，使其提高科技含量、升级上档、扩大规模。要进一步引进和发展建筑瓷、卫生瓷、工业瓷等高档陶瓷项目，加强新产品开发，推动陶瓷产业向高附加值、高技术含量的品牌化方向发展。要以全国工业固废综合利用示范基地为平台，加快推进以粉煤灰、煤矸石、活性石灰和脱硫石膏为原料的材料工业发展，推动保温耐火材料、地板、家具板材、提取超细纤维等领域的研发及工业化生产。要利用我市煤炭产业优势，引进和发展高端装备制造，特别是能源装备制造和矿山装备再制造，支持中煤平朔宇辰、中煤四达、繁盛昇等企业提升维修装备和工控信息化水平。要争取国家支持，扩大风电、光伏发电、生物质能发电等新能源电力规模，建设先进技术光伏发电示范基地，力争新能源装机规模比重稳定在30%以上。抓好平鲁的虎头山风电场三期、刘家窑风电场一期、白玉山风电场二期、败虎堡风电场二期、红石峁风电场和2万千瓦光伏发电项目，以及右玉的小五台风力发电一期扩容项目、垃圾焚烧发电厂等装机容量70.75万千瓦的8个在建新能源发电项目。与此同时，要大力推动已开工的山阴昱光二期、同煤朔南、平朔木瓜界低热值煤发电厂加快建设进度。要积极支持新能源汽车发展，加快城乡区间电动乘用车试点建设。医药产业重点抓好玉龙化工原料药和农药中间体生产线、中诺药业年产2000吨甲磺胺生产线建设。

　　发展战略性新兴产业。紧跟5G时代，充分发挥我市气候冷凉、电力充沛等优势，积极发展新一代信息技术产业。推动山西北方北斗卫星导航项目加快建设。构建大数据产业框架，进一步开发云计算、物联网、移动互联网产业，推动大数据与制造业、现代农业、服务业逐步融合，以信息化促进新型工业化、新型城镇化和农业现代化。

　　煤炭资源清洁高效利用。顺应能源革命要求，加快传统产业与新技术、新工艺、新模式相互嫁接，明确方向，找准路径，延伸煤炭产业链，促进煤基产业绿色清洁高效循环发展。全力推动已签署协议的阳煤集团年产200万吨低阶煤分质清洁利用多联产、松蓝化工年产2×40万吨丙烯酸及脂类、普大集团年产30万吨洁净型煤、神雾集团年产40万吨聚乙烯、中煤平朔年产75万吨煤制烯烃等一批煤化工项目落地。

　　大力发展民营经济。要培育一批产业方向好、企业成长性好的民营企业逐步做大做强。支持一批民办煤矿发展非煤项目，推动民营经济实体进行农业产业开发、旅游产业开发和发展三产服务业。要进一步放宽民营经济发展新产业、新业态的市场准入，建立公平竞争的审查制度，按照统一市场、公平竞争的标准，清理和废除对民营经济的歧视性规定，保障非公有制经济与公有制经济一样，平等使用生产要素、公平参与市场竞争。推动基础设施、公共服务、金融、能源等领域向民间资本开放。要进一步落实国家产权保护制度，依法保障非公有制企业与公有制企业享有平等的财产权。

　　实施科技创新和人才强市战略。科技创新是转型发展的第一动力，人才战略是转型发展的核心要求。我们要实施科技创新战略，以企业创新为主体，引导企业加大研发投入，建立企业技术中心，发展高新技术项目。进一步加强北大固废研发中心和中北大学朔州校区的合作，做好山西省生态农牧研究院挂牌组建工作。继续办好亚洲粉煤灰及脱硫石膏处理与利用技术国际交流大会，打造具有国际影响力的新技术新成果展示平台。要实施人才强市战略，制定一流的人才政策，构建全新的人才自主流动机制，健全“不求所有、但求所用”的人才引进机制，吸引各类高层次人才创新创业。依托重大科研项目、重点实验室等人才培养载体，着力抓好高层次创新型科技人才的培养。鼓励朔州企业走出去，到京津地区与全国知名高等院校、科研院所进行深度合作，培育一批管理人才，转化一批科技成果。

　　全面推动产业转型、经济转型，是解决我市发展中面临的一系列矛盾和问题的主要抓手，是实现我市全面小康、振兴崛起的根本出路。我们一定要抓住难得的历史机遇，主动转型、创新转型、深度转型、全面转型，大力弘扬右玉精神，以功成不必在我的历史责任感，不懈努力，久久为功，积小胜为大胜，就一定能够打好打胜转型发展这场硬仗！

　　（三）加快城镇化建设步伐。按照市域范围内“一核两区两轴”的城镇化总体规划和空间布局，构建市城区、大县城、新型城镇、美丽乡村“四位一体”城镇化建设格局。

　　加快市城区发展步伐。着力推动市城区基础设施全面配套建设、公共服务优质化建设、经济发展“首位度”建设。今后市城区建设要确立这样五个新理念，即：牢固树立规划至上、生态宜居、精致建设、限制高层、体现特色的理念，把市城区建设成为具有鲜明特色的“园林式”城市。要大规模开展城市植绿种树，使城市绿化覆盖率达到45%以上，到“十三五”期末城市人均绿地面积达到全国最好水平。从今年起，我们要投入较大的力量，加强城市发展规划，成立朔州市城市规划勘察设计院，聘请国内知名的城市规划勘察设计单位，对我市城市总体规划进行调整完善。今明两年，要完成市城区的40多个专项规划，为长远发展奠定好的基础。

　　抓好城市基础设施全面配套建设。今明两年要重点实施十大类城建工程项目，总投资103亿元，包括路桥工程、绿化工程、景观工程、供水工程、污水工程、垃圾处理工程、七里河和恢河综合治理工程、地下综合管廊工程、公共服务工程、商业网点工程等。今年新开工51项重点工程，当年完成投资50多亿元。重点抓好恢河大桥、“三路四桥”、高铁快速通道、地下综合管廊、怡西路北延、古北街东延、市府街东延、建设路北延、四水厂、生活垃圾第三填埋场等工程。

　　实施公共服务优质化建设。要提升公共服务水平，着力建好涉及教育事业、卫生事业、文化事业、商贸物流业的配套工程。要推进飞机场、高铁站、朔州市第一人民医院、市一中、体育中心、商业综合体、科技馆、公交调度中心等一批公共服务设施建设。加快推进棚户区和城中村改造，开工6750套，基本建成5000套。在抓好大工程大项目的同时，也要抓好一批城市便民服务设施建设工程。新建7个居民菜市场，新建、改建一批示范性普惠幼儿园，改造完成12条小街小巷。完善市城区公共交通网络，投放150辆新能源公交车，新改建113个公交候车厅，全面开通15条公交线路。投放3000辆公共自行车，方便群众出行。持续开展市城区市容市貌“五乱”整治和卫生清洁两个“专项行动”。深入推进“五城联创”。巩固提升国家园林城市、全国双拥模范城创建成果，卫生城市、环保模范城市、文明城市要在已经获得省级称号的基础上，加快争取国家级称号。

　　健全城镇化推进机制。完善城乡一体化建设和发展的体制机制，推进城乡“规划编制、基础设施、公共服务、产业布局、制度安排”五个一体化。深入实施大县城战略，加快全市城镇群建设，重点抓好十个特色示范镇，提高城镇人口承载能力。合理安排全市范围内城镇建设、农田保护、产业集聚、村落分布、生态涵养等空间布局，推动水电路气等基础设施城乡联网、共建共享，促进各种资源要素在城乡之间协调配置，推进教育、医疗、文化、社保等基本公共服务资源向基层倾斜、均衡配置。

　　（四）促进农业增效农民增收。农业和农村经济发展要紧紧围绕农民收入稳定增长这个中心环节，牢牢把握农业供给侧结构性改革这条主线，把草牧业发展作为农业经济的主战场，努力打造全国草牧业发展示范市、全国农牧交错带结构调整先行区。

　　深化农业供给侧结构性改革。按照“稳粮优经扩饲”的思路，实施种植业结构调整优化工程、草牧业试验示范工程、耕地综合生产能力提升工程。突出抓好100万亩高产玉米、100万亩饲用玉米、100万亩小杂粮、100万亩经济作物基地建设。粮食产量稳定在10亿公斤左右。把发展草牧业作为农业供给侧结构性改革的主抓手，完成耕地种草77万亩。启动朔州生态畜牧产业集聚区建设，重点打造好1000平方公里的怀仁现代肉羊产业集聚区和1000平方公里的山阴、应县现代奶牛产业集聚区。奶牛存栏达到19万头，肉羊饲养量达到600万只。积极做好全国草牧业发展现场会和全国杂粮全产业链现场会筹备工作。进一步做大做强农产品加工业，培育古城乳业、山西雅士利、怀仁皮革城、应县恒天然等龙头企业。全市农产品加工企业销售收入达到205亿元，同比增长6.8%。

　　积极发展农业社会化服务。着力构建农业生产服务体系、农业科技创新和技术推广体系、农产品市场流通体系、农业保险体系、农业信息化服务体系等，进一步完善产前、产中、产后全程社会化服务。加快基层农技推广改革，培育多种形式的农业经营性服务组织。探索开展政府向农业经营性服务组织购买公益性服务试点，鼓励和支持工商资本发展现代种养业和农产品加工流通业，提供农业社会化服务。

　　加强美丽宜居乡村建设。坚持因地制宜、突出特色，分类指导、分步实施，大力实施以“完善提质、农民安居、环境整治、宜居示范”为主的农村人居环境改善四大工程，加强农村基础设施建设。重点抓好垃圾治理、污水治理“两治”和改水、改厕、改路、改房、改能“五改”，改善农村生产生活条件和公共服务。全面完成3765户农村危房改造，完成30个村庄的农村气化工作，启动实施100个市级美丽乡村示范点建设，积极发展休闲度假、养生养老、旅游观光、农耕体验、创意农业、乡村手工艺等农村新业态。高度重视贫困地区和边远山区人口大量外流的“空壳村”的开发和建设问题，积极探索宅基地置换建设用地的模式，引进战略投资者，组织实施移民并村工程。

　　农业和农村经济是国民经济的基础。我们不能因为农业的比例小了就忽视农业，不能因为农村人口少了就忽视农村，而是要强化对农业基础地位的认识，深刻认识我市农业农村经济发展的巨大潜力。只要我们紧紧抓住农业供给侧结构性改革这个关键，农业种植结构就会发生深刻变化；只要我们紧紧抓住转型发展这个关键，农业的比较效益就会大大提高；只要我们紧紧抓住深化农村改革这个关键，农业农村的活力就会不断增强。一句话，农业和农村经济发展前途光明，前景灿烂！

　　（五）加快发展服务业。

　　促进金融业做大做强。发展普惠金融、绿色金融、创新金融。完成朔州农商行筹建工作，力争上半年挂牌。启动怀仁县农村信用联社改制农商行工作。用好总规模为200亿元的建设银行经济发展基金、浦发银行城市发展基金，加快推进市投资建设开发公司企业债发行，切实提高政府投资能力、财政保障能力、企业融资能力。积极引进更多金融机构，不断提升金融服务地方经济发展水平。要培育资本市场，鼓励支持企业上市，加大企业直接融资。

　　加快文化旅游产业成长发展。深入挖掘整理历史文化资源，高起点谋划文化旅游产业。围绕整体提高朔州在国际国内的文化美誉度，全力整合和打造好应县木塔、右玉精神、安太堡露天煤矿、峙峪猎马人、广武明长城、金沙滩古战场这六个文化名片，深入挖掘其深厚的历史文化内涵，进一步提高和拓展知名度，使其形成牵动朔州产业发展的龙头，成为吸引国内外游客的金字招牌。要推出40个重点历史文化资源开发项目，在精心策划的基础上，广泛开展招商，引进战略合作投资者，进行文化产业开发和文化旅游开发。要深入挖掘整理200个历史文化题材，形成传承地域优秀文化的典籍，部分题材可延伸为影视剧作品。要积极推进应县木塔申报世界文化遗产工作。要重点做好右玉精神这个文化名片的宣传，配合省宣传文化部门完成以右玉精神为题材的大型音乐舞蹈史诗、电视连续剧和话剧三大作品，在北京组织搞好右玉精神研讨会和“塞上绿洲”油画摄影展两大活动，支持右玉干部学院建设，进一步扩大右玉精神的知名度。

　　发展生产性服务业。要进一步发展城市商业、仓储、物流等生产性服务业。重点围绕矿山开采、煤炭外运、机械维修，发展矿山物流、交通运输、维修保养等服务业，带动全市生产性服务业整体快速发展。建设煤炭交易市场和煤炭外销内陆港。目前平鲁区、中煤集团和河北港口集团正在洽谈的内陆港项目建设，是我市服务业发展的重大工程。一期工程规划建设煤炭信息、物流金融、交易结算“三大平台”，项目建成后，既可提高煤炭产业效益，又可规范煤炭营销市场秩序。

　　继续发展好消费性服务业。要积极引进商业连锁店、大型超市等消费性服务业。鼓励建设家政服务、生鲜超市、便民修理等社区商业服务网络，完善社区商业服务。与国内外大型物流公司、快递企业合作，在山西雁门关农贸物流园区设立现代化电商物流中心和公共智能仓储配送中心。发展文化、体育、休闲、养老、健康等幸福产业。

　　（六）全面深化改革。改革是发展的必由之路。要围绕事关全局的一些关键性改革，以转型综改区建设为统领和切入点，以重大改革、重大事项和重大项目为支撑，大胆探索、先行先试，用改革破解经济社会发展难题。

　　推进供给侧结构性改革。落实好“三去一降一补”任务，力争完成1360万吨的去产能任务。探索建立购租并举的住房制度，用足用好住房公积金，多措并举促进商品房去库存。支持有条件的企业依法实施债转股，降低企业综合成本，减轻企业负担。

　　深化农村改革。基本完成农村土地承包经营权确权登记颁证工作，年内市县农村产权交易中心全部挂牌运营。开展农村集体“三资”清产核资，稳步推进农村集体产权制度改革。积极发展家庭农场、专业大户、农民合作社、农业产业化龙头企业等新型农业经营主体，通过经营权流转、股份合作、代耕代种、土地托管等多种方式，促进土地规模经营。进一步加大补贴力度，发展特色农产品保险，并实行以奖代补，不断完善农业自然风险和市场风险的分散机制。

　　加快电力体制改革步伐。扩大直供电范围，探索工业园区与发电企业直接交易的体制机制，探索微电网运行模式，力争将我市列为全省市域电力体制改革先行先试市。落实煤电一体化企业享受厂用电价政策，构建煤、电、网、载能产业协同发展新格局。推动中煤平朔、中煤华昱增量配电申请获批，并列入全省区域电网运营试点，推动润臻公司列入固废综合利用企业厂用电试点。

　　抓好开发区改革创新发展。开发区改革创新发展是经济转型的重要抓手，是战略突破口。以创建国家级、省级开发区为目标，围绕“整合、改制、扩区、调规”和“三化三制”内部改革，加大开发区改革创新发展。按照“一市一国家级开发区，一县一省级开发区”的目标要求，朔州经济开发区力争用2—3年时间申报国家级开发区，其它五个园区尽快打造成省级开发区。

　　加快旅游景区（景点）体制机制改革。围绕管理权、经营权“两权”分离，进一步理顺管理体制、经营机制和利益关系，推进朔城区崇福寺、山阴县广武、怀仁县金沙滩、应县木塔、右玉县杀虎口景区建立现代企业法人治理机构。完成市县旅发委组建工作，加强旅游产业发展统筹协调。组建朔州旅游产业投资公司，引入战略投资者，包装一批旅游项目，进一步提升景区（景点）的核心竞争力。

　　深化商事制度改革。深入推进“五证合一”改革，继续深化“先照后证”和工商登记便利化改革，力争全年市场主体总量同比增长8%，私营企业数量同比增长10%以上。加强事中事后监管，落实“双随机一公开”机制，构建新型市场监管体系，营造良好的市场环境。

　　（七）进一步提升对外开放水平。我们要进一步扩大对外开放，立足国际国内两个市场、两种资源，积极跟进国家开放战略，坚持引资引技引智并重，努力拓展发展新空间、打造发展新优势。

　　不断拓展开放领域。积极参与京津冀一体化，主动融入环渤海经济圈，在产业发展、招才引智、科技创新、基础设施、能源供应、文化旅游等方面加强合作，把我市建成京津冀清洁能源供应基地、旅游避暑休闲养老的“后花园”和优质农产品供应基地。充分利用煤电下江南的机遇，引深与长三角和珠三角地区合作，承接相关产业转移。

　　用好各类招商平台。组织参加好省政府组织的“山西品牌中华行”、“山西品牌丝路行”、“山西品牌网上行”等招商引资活动，力争签约一批大项目、好项目。做好名优特产品外埠展销活动，举办朔州特色商品“华夏行”活动。大力发展电子商务，通过乐村淘、淘宝、苏宁易购等电商，将朔州名优农产品推向全国。培育右玉图远、山西晋坤、诺成制药等一批拥有自主品牌的重点出口企业。

　　高效精准招商引资。围绕先进装备制造、高端陶瓷、新能源、新材料、文化旅游、特色农业、生物制药和现代服务业等战略性新兴产业，包装策划一批转型项目。推进招商引资体制机制改革，实施专业招商、以商招商、网络招商、委托招商等，努力引进更多好项目、大项目。要改进和完善招商引资考核体系，把招商引资工作纳入目标责任考核范围，重点考核签约项目的开工率、资金到位率，并加大考核权重。组织好京津冀、长三角、珠三角等点对点招商活动，力争各县区项目签约额是重点工程投资额的3倍以上，全市签约总额突破1500亿元。

　　（八）大力发展民生和社会事业。牢固树立以人民为中心的发展思想，大力发展社会事业，推进基本公共服务均等化。财政支出要向民生倾斜、向农村倾斜、向困难群体倾斜，让更多的人享受公共财政的阳光，不断提升人民群众的获得感和幸福感。

　　加快发展教育事业。以办好人民群众满意教育为目标，大力推进义务教育均衡发展。进一步调整和优化教育布局，新建、扩建、改建一批中小学校，年内开工建设市一中新校区，新建市七小。进一步加强农村中小学布局调整，培育更多的农村教育优质资源。深化教育教学改革，在“中小学就近划片入学、优秀教师和校长校际交流、优质高中指标下校、电脑均衡编班”等改革上有新的突破。下功夫提高普通高中教育质量，提高高中升学率。大力发展职业技术教育，扩大职业中学的招生计划，普职学生比例逐步达到1︰1。规范民办教育，促进民办教育健康有序发展。市本级要率先抓好市教育行政部门直管的中小学校。既要加大基础建设投入，又要尽快提高教学质量水平，为全市作出示范。

　　发展医疗卫生事业。贯彻落实全国卫生与健康大会精神，推进“健康朔州”建设。加大优质资源下沉力度，推动经费、设备、科技、人员、技术、管理等各类优质资源向基层流动，提升计生服务能力和基层医疗卫生机构服务能力。探索公立医院薪酬制度改革和医疗、卫生、计生、疾控“四位一体”网格化管理模式。鼓励和引导社会资本举办医疗机构。招录和培养更多专科医生，加强医疗队伍建设。

　　继续提高社会保障水平。做好高校毕业生、去产能下岗职工、农村转移劳动力、城镇失业人员、退役军人等群体的就业工作。巩固全民参保登记试点成果，扎实推进“五险”参保扩面。稳步推进机关事业单位养老保险制度改革，整合城乡居民基本医疗保险制度。继续提高社会救助水平，促进社会福利事业发展。

　　促进文化体育事业发展。继续巩固国家公共文化服务体系示范区建设，深化文化单位管理体制改革，建立既节约又运转高效的机制，全面提升图书馆、文化馆、博物馆、乡镇综合文化站使用效益。推进市级主流媒体融合发展，打造新型主流媒体。实施文化惠民工程，开展送戏下乡、周末大舞台等活动。加强文物和非物质文化遗产保护工作，加强古长城保护。加强档案工作，做好志书和年鉴修编，发挥资政、存史、育人作用。推动全民健身运动，增强人民体质。

　　坚决打好打胜脱贫攻坚战。今年我市确定的脱贫目标是，82个贫困村、19193名贫困人口完成减贫任务，特别是平鲁区、山阴县2个省级贫困县（区）要整体“摘帽”。我们要按照“六个精准”、“五个一批”的要求，坚持精准扶贫、精准脱贫，突出抓好产业项目扶贫、就业安置增收、金融信贷扶贫、扶智助教治贫、医疗救助济贫、发展水利扶贫、生态补偿减贫、移民搬迁安居、基础设施改善、政策兜底保障等“十大措施”。要严格退出机制，绝不能搞数字脱贫、虚假脱贫，过好群众满意关，经得起历史检验。

　　办好民生事业，既是人民群众的期盼，也是我们的职责所在。我们一定要把为人民群众所办的实事办实、好事办好，把全市人民干事创业的力量凝聚到全面建成小康社会的伟大实践中！

　　（九）加强生态文明建设。树立“绿水青山就是金山银山”的理念，持之以恒推进环境保护和生态建设。

　　坚持植绿兴水并举。重点实施朔城区南山生态综合治理一期、平鲁区高石庄造林绿化、山阴县偏岭荒山造林、怀仁县环城绿化、应县白马石荒山造林、右玉县大南山荒山造林“六大工程”，完成营造林12.3万亩。狠抓林木资源管护，从今年起对全市550万亩林木进行全域管护。积极推进退耕还林，对上一轮退耕还林补助延长5年，启动新一轮退耕还林。同时，进行生态建设的“二次创业”，兼顾生态效益与经济效益，加大经济林种植力度，实现绿水青山向金山银山转变。加强农田水利建设，实施好灌区节水改造、膜下滴灌、水土流失治理等工程，完成农田灌溉面积227万亩、治理水土流失面积30万亩。

　　强化环境污染治理。今年要继续打好大气、水、土壤污染防治等环境治理的“三大战役”。在大气污染防治上，要把控煤、治污、管车、降尘等重要措施落地落实，改善大气质量。大力推广集中供热和煤改气、煤改电，促进燃煤电厂超低排放升级改造，积极推进朔南、木瓜界、华昱二期热电联供项目，坚决取缔集中供热范围内10蒸吨以下燃煤锅炉；依法关停不达环保要求及无证经营的小型煤场、洗煤厂、石灰窑等土小企业；按期完成陶瓷和其它行业煤气发生炉达标改造。在水污染治理上，加强工业集聚区水污染治理，加大城市污水收集管网建设，逐步实现雨污分流；实施最严格的水资源管理制度，落实河长制。启动土壤污染状况详查，推动土壤污染治理与修复技术应用试点和土壤污染综合防治先行区建设。

　　开展生态治理修复。统筹实施采煤沉陷区土地复垦整理、矿山生态环境恢复治理工程，积极推进七里河综合治理工程。特别是要规划实施好已列入国家水利部重点项目的桑干河流域综合治理和生态修复工程。该项治理工程在朔州境内治理区域涉及朔城区、山阴县、应县、怀仁县四县区，全长131.5公里，总投资34亿元。这是朔州生态环境建设史上一项造福子孙后代的战略工程。工程完成后，将使朔州境内特别是桑干河流域内水环境和生态环境发生根本性的变化。我们要按照工程规划的“一河两区三带”建设的总体布局和设计，动员和组织全市人民全方位参与工程建设，支持工程建设，确保工程尽早发挥效益。

　　（十）坚持不懈抓好安全生产和社会稳定工作。

　　压实安全生产责任。认真开展“安全责任落实年”活动，坚持“党政同责、一岗双责、企业（单位）主责、失职追责”，按照“管行业必须管安全、管业务必须管安全、管生产经营必须管安全”和谁主管谁负责的要求，不折不扣把党委政府领导责任、部门监管责任和企业主体责任落到实处。

　　持续开展以煤矿安全隐患为重点的专项整治。深刻汲取中煤担水沟煤矿“1·17”重大顶板事故教训，在煤矿、非煤矿山、道路交通、危险化学品、燃气管道、建筑施工、人员密集场所等行业领域开展安全生产隐患大排查大整治。对重大隐患实行挂牌督办、限期整改，整改无望的予以关闭，对非法生产经营建设行为坚决打击取缔。

　　加强安全工作的基层基础建设。在重点领域强制推进安全生产标准化建设，开展反“三违”专项行动，加强企业生产一线、作业现场的安全生产管理。加大安全生产投入，提高安全技术装备水平。加强乡镇执法力量，完善开发区、工业园区等功能区的安全生产监管体制。

　　全力维护社会稳定。深入开展平安朔州建设活动，保持“打黑除恶”高压态势，严防暴力恐怖活动，完善立体化社会治安防控体系，提高人民群众安全感。加强新形势下国防动员、国防教育和后备力量建设，积极支持军队规模结构和力量编成改革，推进军民融合深度发展。实施质量强市战略，促进质量安全根本改善。加强食品药品监管，加快建立重要产品追溯体系。要创新信访工作机制，有效调处各类矛盾纠纷，维护社会和谐稳定。四、加强政府自身建设

　　面对严峻复杂的经济形势，面对转型综改的繁重任务，各级政府必须适应新形势新任务的要求，进一步提高执政能力和执政水平。

　　（一）加强思想政治建设。深入学习贯彻习近平总书记系列重要讲话精神和治国理政新理念新思想新战略，切实增强政治意识、大局意识、核心意识和看齐意识，始终在思想上、政治上、行动上同党中央保持高度一致。深入贯彻党的基本路线，坚持改革开放，坚持以经济建设为中心。牢固确立全心全意为人民服务的宗旨，认真贯彻民主集中制，真抓实干，敢于担当，努力建设人民满意的政府。

　　（二）加强履职能力建设。面对异常艰巨复杂的改革发展任务，各级政府必须努力提高履职尽责的能力。要增强领导发展的能力。切实把抓发展作为第一要务，不断提高新形势下领导经济工作的能力。要增强执行政策的能力。深入贯彻落实党的路线、方针、政策，紧密联系朔州改革发展的实际，创造性地开展工作。要增强解决问题的能力。善于在尖锐复杂的发展形势中抓主要矛盾、解决突出问题。要增强改革创新的能力。解放思想，大胆创新，勇于探索，用改革创新的办法推动经济社会发展。要增强推动落实的能力。以踏石留印、抓铁有痕的劲头，推动工作落实。

　　（三）加强法治政府建设。要在各级政府中进一步强化法治思维和法治意识，切实提高依法行政、依法办事的水平，按照“法定职责必须为，法无授权不可为”建设法治政府的要求，把政府各项工作全面纳入法治轨道。要推进决策科学化、民主化、法制化，建立重大行政决策合法性审查机制、终身责任追究制及责任倒查机制。要认真执行人大及其常委会的决议决定，定期向人大及其常委会报告工作，主动接受人大监督。要认真办理人大代表建议、政协委员提案、人民群众来信来访，做到事事有回音，件件有落实。

　　（四）加强行政效能建设。按照建设服务型政府的要求，深化“放管服效”改革，持续推进简政放权、放管结合、优化服务，不断提高政府工作效能。要深化行政审批制度改革，继续做好审批事项承接、取消、下放、调整工作。进一步提升政务服务中心和公共资源交易平台服务功能。深入落实好“13710”工作制度，尽快在市县乡三级政府全面建立电子督办平台。要进一步解决好一些干部工作中存在的不作为、慢作为、乱作为等问题；坚决整治推诿扯皮、敷衍塞责等懒政怠政行为，不断提高政务服务质量和水平。要健全完善工作目标考核机制，把全年工作任务分解到各部门，做到任务到人，责任到人，严明奖惩，严格问责。

　　（五）加强政府廉政建设。政府党组要切实扛起全面从严治党主体责任，认真落实“一岗双责”，不断加强政府系统党的建设。要严守党的政治纪律，严肃党内政治生活，坚决把纪律和规矩挺在前面，强化对行政权力的监督和制约，对公共资金、国有资产、国有资源和领导干部履行经济责任实行审计全覆盖，切实把权力关进制度的笼子。要严格执行中央八项规定和国务院“约法三章”精神，坚决防止“四风”反弹回潮。牢固树立“过紧日子”思想，厉行勤俭节约，反对铺张浪费，把有限的资金用在改善民生、推动发展上。要深入开展政府系统党风廉政建设和反腐败斗争，努力形成干部清正、政府清廉的良好局面！

　　各位代表，我们正处于转型发展、创新发展、全面小康的关键历史时期。全市人民对经济走出困境、实现稳定发展充满了期待！对加快发展、振兴崛起充满了期待！让我们紧密团结在以习近平同志为核心的党中央周围，在中共朔州市委的坚强领导下，充分调动各方面的积极性，汇聚起推动发展的强大力量，撸起袖子加油干，以优异的成绩迎接党的十九大胜利召开！
